# Development and evaluation of a propolis, tea tree oil, and jojoba oil nanoemulgel with enhanced antioxidant, anti-inflammatory, and wound-healing activities

**DOI:** 10.1038/s41598-026-50846-y

**Published:** 2026-05-25

**Authors:** Mariam H. Saad, Soha M. Kandil, A. Farid, Gehan Safwat, Ayman A. Diab, Mohamed Taha

**Affiliations:** 1 Faculty of Biotechnology, October University for Modern Sciences and Arts (MSA), Giza, 12566 Egypt; 2 Department of Pharmaceutics and Drug Manufacturing, Faculty of Pharmacy, Modern University for Technology & Information (MTI), Cairo, 12055 Egypt; 3 Nano Gate Company, 9254 Hoda shaarawy, Al Abageyah, El Mukkatam, Cairo, 11571 Egypt; 4 Electronic and Magnetic Materials Department, Advanced Materials Division, Central Metallurgical Research and Development Institute (CMRDI), Cairo, 11421 Egypt

**Keywords:** Propolis, Nanoemulgel, Wound healing, Anti-inflammatory, Antioxidant, Biotechnology, Cancer, Drug discovery, Medical research

## Abstract

The wound healing’s acceleration depends on developing an innovative therapeutic strategy that reduces inflammation, regulates oxidative stress, promotes fibroblast proliferation, and collagen deposition. This study aimed to prepare and evaluate a green nanoemulsion-based formulation containing propolis, tea tree, and jojoba oils to integrate their biological activities for enhanced wound healing efficacy. Nanoemulsion formulations were prepared via a low-energy emulsification approach and characterized by zeta potential, hydrodynamic size (DLS), polydispersity index (PDI), and transmission electron microscopy (TEM). The in vitro antioxidant and anti-inflammatory activities were assessed. In addition, cytotoxicity was assessed using the MTT colorimetric assay against the HSF-1 normal skin cell line. Additionally, NO and *IL-1β* levels were measured on NHDF-Ad cells. A propolis nanoemulgel (PNEG) was produced using Carbopol 940 as a gelling agent. The in vivo wound healing efficacy of PNEG was tested by evaluating wound closure rate, malondialdehyde, superoxide dismutase, tumor necrosis factor-alpha, and histopathological alterations in healed tissues. The obtained results demonstrated that, the optimized formula displayed a zeta potential of − 28.6 ± 1.91 mV, a DLS of 187.8 ± 3.29 nm, and a 50% cytotoxic concentration (CC_50_) of 24.82 ± 0.16% and showed a significant antioxidant and anti-inflammatory activities. Furthermore, NO and *IL-1β* levels were significantly decreased by 35% and 40%, respectively, demonstrating potent anti-inflammatory activity. Additionally, the therapeutic activity was associated with inhibition of *IL-1β*/NO-driven inflammatory signaling, suppression of *NF-κB*, activation of the *NRF2/HO-1* antioxidant axis, and modulation of *BAX/Bcl-2* apoptotic pathways. The in vivo results indicated that PNEG accelerates wound closure significantly compared with both positive and negative control groups. SOD level increased significantly (*p* < 0.05), whereas MDA and TNF-α levels decreased compared with the control groups (*p* < 0.05). Furthermore, histopathological examination illustrated infiltration reduction of inflammatory cells, increased collagen deposition, and fast epithelialization. The developed green formula, resulting from both the low-energy, environmentally friendly preparation method and the natural origin of its constituents, was demonstrated to be an antioxidant and inflammatory and to accelerate wound healing, suggesting it could be used as a natural formula for topical wound healing application.

**Supplementary Information:**

The online version contains supplementary material available at 10.1038/s41598-026-50846-y.

## Introduction

The human body’s largest organ is the skin, it has served a variety of purposes, including preserving bodily fluid balance, controlling body temperature, warding off microbes, avoiding microbial diseases, the skin’s surface is home to a diverse range of bacteria, such as *non-pathogenic mycobacteria*,* streptococci*,* staphylococci*,* and candidiasis*^1,2^. Damaged skin is more susceptible to diseases from the outside environment, and microbes can grow around the wound and spread infection^3^. Apart from this, the main target for dermal/transdermal medication administration is the skin care. It has attracted a lot of study interest as a non-invasive substitute for conventional oral or injectable medication administration because of its sizable, readily accessible surface area^4^. Burns, trauma, chronic diseases, and surgical wounds can all result in skin damage that impairs function and is an acute concern for healthcare systems. Because of their complex pathophysiology, treating chronic wounds is more challenging than treating acute ones^5,6^. Skin wound healing (WH) is a crucial process in terms of re-establishing barrier integrity to avoid further injury or infection. It involves different types of cells and mediators, including leukocytes, neutrophil granulocytes, and chemokines^7^. The four overlapping stages of hemostasis, inflammation, proliferation, and tissue remodeling can be used to describe WH, which is a dynamic but highly regulated process. The coagulation cascade during the hemostasis phase triggers the transient wound matrix. This is followed in the inflammatory phase by the requirement of an innate immune response to decompose and remove tissue and pathogen debris. Numerous disadvantages of older wound care techniques include the inability of medications to penetrate inner skin tissues and the development of microbial resistance with prolonged use of antibiotics^8^. Novel drug delivery systems are designed to improve the therapeutic efficacy and safety of drugs by enhancing their bioavailability, stability, and targeted delivery. These systems, including nanoparticles, liposomes^9^, nanoemulsion (NE)^10^, and polymeric carriers^11–14^, enable controlled and sustained drug release at the desired site of action^15^. By minimizing systemic exposure, novel carriers can significantly reduce adverse effects and required dosing frequency^16^. Overall, advanced drug delivery approaches play a critical role in optimizing clinical outcomes and patient compliance. Antifungal, antibacterial, and anti-inflammatory properties are among the most prominent of the physiologically active components found in essential oils, which are recognized as natural compounds^17^.

Tea tree oil (TTO) has a broad-antimicrobial spectrum against bacterial, viral, fungal, and protozoan infections that affect the skin and mucous membranes in addition to its potent antioxidant activity due to its distinct pharmacological properties, which include immunological modulation, anti-inflammatory, and anti-tumor activities. This makes TTO suitable for use in WH applications^18^. However, TTO’s hydrophobicity, volatility, instability in the presence of light or oxygen, and formulation challenges limit its usage in pharmaceutical production. Encapsulating TTO at the nanoscale provides an efficient method of enhancing its physical stability by protecting the active chemicals from environmental reactivity and volatilization^19^. Furthermore, jojoba oil, which is produced from *Simmondsia chinensis* seeds, has anti-inflammatory qualities, and TGFβ1 overexpression has been connected to enhanced production of extracellular matrix components^20^. Because of its biological features, which include antimicrobial, anti-inflammatory, antipyretic, analgesic, antioxidant, and anti-hyperglycemic effects, jojoba oil is currently used in a wide range of bio-medical industries, including cosmetics and treatments that cure skin disorders (psoriasis and acne)^21^. Additionally, it has been used to superficial wounds to speed up their recovery. Jojoba oil has pharmacological value as an adjuvant in WH treatments because it not only encourages collagen synthesis in fibroblasts but also improves wound closure by acting on keratinocytes and fibroblasts^22^. There is a renaissance in apitherapy wound dressing, especially those related to honey and propolis and acted with high activity. Propolis is a resinous substance, generally dark yellow, orange, brown, or tan in color^23^. Furthermore, due to propolis’s excellent antiseptic efficacy, antimicrobial properties, and anti-inflammatory activity^24^ it accelerates the contraction and accelerate tissue healing phases, which minimize the time it takes for wounds to heal. Recent apitherapy product nanofabrication WH offer the benefits of short treatment duration, facile application, and potency with various apitherapy product activities, particularly those polymeric networks^25^. Because they are also highly lipophilic, oil-in-water (o/w) nano-emulsions are widely used in the pharmaceutical sector to distribute lipophilic active substances that readily pass through biological membranes^26^. Due to their many advantageous characteristics, NEs have been recognized as a better choice for improved WH, resulting to a development in their use in wound management. The main advantages of using NEs as drug delivery vehicles in the management and treatment of WH include high drug loading capacity, improved drug solubility and bioavailability, relatively simple preparation and scale-up, controlled drug release, and defense against enzymatic degradation^27^. Despite extensive studies on individual herbal nanoemulsions for WH, there remains a lack of systematic investigations into multi-component nanoemulgel systems combining propolis, tea tree oil, and jojoba oil, particularly regarding their synergistic antioxidant, anti-inflammatory, and wound-healing effects within a single topical formulation. A novel nanoemulgel’s pH, stability, biological adhesion, diffusion, droplet size, polydispersity index (PDI), zeta potential, and in vitro and in vivo protein screening are some of the characteristics that should be examined to assess the prepared nanoformulations^28^.

Hence, this study aims to integrate the biological properties of propolis, tea tree oil, and jojoba oil to develop, characterize, and evaluate a propolis-based nanoemulgel (NEG) to achieve the delivery advantages, high thermodynamic stability, controlled drug release. The formulations were assessed for their physicochemical characterizations, including zeta potential, DLS, PDI, and TEM, in addition to anti-inflammatory and antioxidant activities as well as, study cellular mechanisms, mechanistic insights, and in vivo wound-healing efficacy in albino rats.

## Experimental work

### Materials

Absolute ethanol was purchased from Merck, Germany. Propolis, Jojoba oil and Tea tree oil were purchased from the local natural plants store (Iemtnan, Cairo 4413045, Egypt); Lecithin was obtained from Advent-India; Tween 80, Span 80, Carbopol 940, Sodium lauryl sulfate (SDS), and triethanolamine (TEA) were purchased from Loba-Chemie-India; MTT, Dimethyl sulfoxide (DMSO), Folin reagent (CN: F9252), and sodium Diclofenac were also purchased from Sigma-Aldrich-USA; L-glutamine, Fetal Bovine serum, HEPES buffer solution, DMEM, and 0.25% Trypsin-EDTA, gentamycin were obtained from Lonza (Belgium). All materials were used without further purification. Double-distilled water (DDH_2_O) was utilized to prepare the solutions.

### Preparation methods

#### Extraction of Propolis by maceration method

Maceration method using Ethanolic solution was used to extract the Propolis with a low wax content and rich in bioactive compounds. Briefly, 10 gm crude grounded Propolis was soaked in 100 ml of (70% V/V) aqueous ethanol solution to extract the highest phenolic compounds, that responsible for the biological activities of propolis^29^. Extraction was carried out at 50 °C with periodical shaking for 24 h. then, it was filtered and double-checked using filter paper and gauze then the ethanol solvent was eliminated completely by using a rotary vacuum evaporator (D-Lab, USA).

#### Preparation of nano-emulsion

According to our previous work^30^, jojoba: tea tree oil nano-emulsions were prepared by a low-energy method. Briefly, the oil phase, Surfactants, and propolis extract were mixed via a magnetic stirrer (Cimarec+, ThermoFisher Scientific, USA) until they were homogenous. After that, using a 50 W input power sonication (VCX 500, Sonics, USA) and homogenization, DDH_2_O was added dropwise to the oil phase mixture. To control the produced thermal effect, the mixture was put in an ice-path, which homogenized the whole system forming oil/water NEs.

Table 2 illustrates the % composition of optimized Jojoba and Tea Tree nano-emulsions. Propolis was loaded using the same methodology by adding it in various concentrations 2.5%, 5% and 10% (w Propolis /w oils) FN-P1, FNP-2, and FN-P3 respectively in oil phase.

#### Propolis Nanoemulgel (F-PN) preparation

According to the previous reports, Carbopol 940 at a concentration of 1% can be utilized as a safe gelling agent for topical applications and has the advantage of extending drug penetration into the skin^31^. To produce a highly viscous solution, 1% w/v of it was first mixed thoroughly with DDH_2_O on a Homogenizer (Dihan HG-15D, South Korea) at room temperature and allowed to sit for 24 h. As a humectant, glycerin was added to create a soothing and smooth effect. After that, 20% optimized NE was evenly distributed throughout a hydrogel system, TEA was used to adjust the pH (6–8). Before it was utilized for in-vivo study, the resulting nano-emulgel was stored at 4 °C.

### GC-Mass analysis

A GC–TSQ mass spectrometer (Thermo Scientific, USA) fitted with a TG-5MS capillary column (30 m × 0.25 mm × 0.25 μm) was used to determine the chemical constituent of propolis extract. Briefly, the oven temperature was set to start at 60 °C and increase to 250 °C at a rate of 5 °C per minute for two minutes, and then to 300 °C for thirty minutes. Temperatures were 270, 200, and 280 °C for the injector, ion source, and transfer line, respectively. The carrier gas was helium (1 mL/min). A 1 µL sample was injected in split mode (4 min solvent delay), and EI mass spectra were recorded at 70 eV over m/z 50–650. Components were identified by matching spectra with NIST14 and WILEY 09 libraries.

### Physicochemical characterization

Zeta potential, dynamic light scattering (DLS), and poly dispersive index (PDI) measurements were performed using the Zetasizer ZS90 (Malvern Instruments, UK). A JEOL JEM-2100 high-resolution transmission electron microscope (HR-TEM) operating at an accelerating voltage of 100 Kv was used to measure the NEs’ particle size and shape. A droplet was placed on a Formvar carbon-coated, 300-mesh copper grid (Ted Pella, USA) to prepare samples from the nano-emulsions for TEM imaging.

### In-vitro release study

Firstly, calibration curve of propolis was calculated as follow. Propolis (5 mg) was dissolved in 100 mL in PBS (pH = 7.4) contains 1% tween 80 to obtain a concentration of 50 µg/mL. and the absorbance was measured at 265.2 nm using a UV visible spectrophotometer (Cary series UV-Vis- NIR, Australia). After that, Serial dilutions were prepared from the stock solution of Propolis in PBS (pH = 7.4) with tween 80 (1% w/w) to produce different concentrations.

To study the release kinetics of propolis NE and propolis NEG, 5 gm of propolis NEs and 5 gm of propolis NEG one was placed into a dialysis bag (MWCO 10:12 kDa) in different two beakers, and then each one was directly immersed into 45 mL of PBS 7.4 contains 1%w/v tween 80 at 37 ◦C. Then, we withdrew 3 ml after 15 min,30 min,45 min,1 h, 2 h,4 h…., etc. to determine the concentration at these interval times. After that, the cumulative release percentage was calculated.

### Ex-vivo study of skin permeation and skin retention

Abdominal skin from male Wistar albino rats (100–150 g) was used for ex vivo permeation studies. At first, the animals were mercifully sacrificed according to ARRIVE guidelines, anesthetized intraperitoneally (IP) with a mixture of xylazine HCl (8 mg/kg) and ketamine HCl (80 mg/kg)^32^, and the abdominal region was carefully shaved. The excised skin was cleaned by removing subcutaneous tissue, wiping the dermal side three times with isopropyl alcohol, and rinsing with double-distilled water. The prepared skin samples were cut into suitable sizes, wrapped in aluminum foil, and stored at − 20 °C until use. The subsequent permeation procedure followed the method described previously^33^.

### Biological assays

#### Anti-inflammatory assay

The albumin denaturation assay was used to evaluate the anti-inflammatory effect of the tested-materials by evaluating their ability to inhibit heat-induced denaturation of albumin. This method is based on the principle that compounds with anti-inflammatory activity can stabilize protein structures and prevent their denaturation, a process commonly associated with inflammation and tissue injury. Bovine serum albumin (BSA; CN: 232936-2) was used as the protein source for this study and the method was performed following the procedure described by Williams et al.^34^.

#### The antioxidant activity

Free radical scavenging activity of the prepared NEs was evaluated using 1, 1- diphenyl-2-picryl-hydrazyl (DPPH) as described in previous works^35,36^.

The percentage inhibition (PI) of the DPPH radical was determined using the following formula:

1 $${\text{DPPH scavenging effect }}\left( \% \right){\text{ }}={\text{ }}{{\mathrm{A}}_0} - {\text{ }}{{\mathrm{A}}_{\mathrm{t}}}/{\text{ }}{{\mathrm{A}}_0} \times {\text{ 1}}00$$

Where *A*_0_; optical absorbance of the control at zero time and *A*_t_; absorbance of the sample + DPPH at time t. The 50% inhibitory concentration (IC_50_) was calculated based om graphic dose response curve plots.

####  In-vitro cytotoxicity assay

##### Cell culture

HSF-1 cells (human foreskin fibroblast skin cell line) were obtained from the American Type Culture Collection (ATCC, Rockville, MD).

##### Propagation of cell lines

The HSF-1cells were propagated in Dulbecco’s modified Eagle’s medium (DMEM) which was provided with 10% heat-inactivated fetal bovine serum (FBS), 1% L-glutamine, HEPES buffer, and 50 µg/mL gentamycin. All cells were maintained at 37 °C in a humidified atmosphere with 5% CO_2_ and were sub-cultured two times a week.

##### Cytotoxicity assay

HSF-1 cells were seeded onto 96-well culture plates at a density of 1 × 10⁴ cells per well in 100 µL of growth media, and they were cultured for 24 h to allow cell conjugation before the NE cytotoxicity was evaluated using the MTT assay. Fresh medium with different concentrations of the NE, prepared as two-fold serial dilutions ranging from 1000 µg/mL to 7.8 µg/mL, was then used to replace the initial medium. After that, the cells were incubated for 24 h at 37 °C in a humidified environment with 5% CO_2_. Control wells received culture medium without NE. After incubation, cell viability was assessed using the MTT colorimetric method as described by Mosmann et al.^37^. Absorbance was measured at 570 nm using a microplate reader (SunRise, TECAN, Inc, USA) to determine the number of viable cells, and the percentage of viability was calculated from:

2 $$\left[ {\left( {{\mathrm{ODt}}/{\mathrm{ODc}}} \right)} \right]{\text{ }} \times {\mathrm{1}}00\%$$

ODt is the mean optical density of wells treated with the NE sample and ODc is the mean optical density of the control cells. The relation between surviving cells and the concentration of NE was plotted to obtain the survival curve for each HSF-1 cell line after treatment. The 50% cytotoxic concentration (CC_50_), the concentration required to cause toxic effects in 50% of intact cells, was calculated from graphic plots of the dose-response curve for each concentration using GraphPad Prism software (San Diego, CA, USA).

#### Assessment of anti-inflammatory response in NHDF-Ad cells

##### Cell Homogenization

After adjusting the cell count to 1 × 106, 350 µL of the lysis buffer was added. Bead-milling in a lysis buffer containing guanidine and thiocyanate disrupted and homogenized the cells. The Tissue Ruptor II (Qiagen, Hilden, Germany), a rotor-stator homogenizer that completely destroys and simultaneously homogenizes samples in the presence of lysis buffer in 15–90 s, depending on the toughness and size of the sample, was used for tissue disruption and homogenization. Then, the mixture is centrifuged for 15 min. at 4000 rpm. Finally, the cell supernatant is collected for RNA extraction.

##### Measurement of protein concentration

Protein concentration is determined using the bicinchoninic acid solution, cat no. B9643, Sigma Aldrich, Milwaukee, Wis., USA assay, which measures colorimetric changes related to amino acids and peptide bonds. First, bovine serum albumin (BSA) standards diluted in the same buffer as the samples are used to create a standard curve. Next, BCA reagent A and reagent B are combined at a 50:1 ratio to generate a working reagent. Add 25–50 µL of each sample and standard to a 96-well plate or test tube, then 200 µL of the working reagent. For thirty minutes, the mixture is incubated at 37 °C. A microplate reader is used to detect absorbance at 562 nm. By comparing the absorbance measurements to the standard curve, each sample’s protein concentration is calculated^38^. For optimal precision, all measurements are carried out in triplicate and samples with high protein concentrations are appropriately diluted. Samples are kept at -20 °C for later use after the results have been examined.

##### Protein expression using ELIZA technique

The Enzyme-Linked Immunosorbent Assay (ELISA) is performed to measure *Human IL-1β (Interleukin 1 Beta)* levels in cell lysate. First, a 96-well ELISA kit *“Human IL-1β (Interleukin 1 Beta)* ELISA Kit Cat No.EH0185, Fine Biotech, Wuhan, Hubei, China. The plate was coated with a capture antibody specific to *IL-1β*. 100 µL of diluted tissue homogenate is added to each well and incubated for 1 hours at 37°C and *IL-1β* levels were measured in accordance with the manufacturer’s instructions. The enzyme concentration is determined using a standard curve, and results are analyzed accordingly^39^.

#### Measurement of Nitric oxide on NHDF-Ad cells

Nitric oxide (NO) production was quantified indirectly by measuring nitrite (NO₂⁻), a stable end product of NO metabolism, using a commercially colorimetric Nitric Oxide Assay Kit, Cat. No. E-BC-K035, **(**Elabscience, Houston, TX, USA)^40^. To eliminate cellular debris, cell culture supernatants were collected 48 h after experimental treatments and centrifuged at 1,000 × g for 10 min at 4 °C. The extracted supernatant was either utilized immediately or kept at -80 °C until analysis. Following the manufacturer’s instructions, the concentration of nitrite was measured using the Griess reaction, which forms a stable purple azo product when nitrite continuously reacts with sulfanilamide and N-(1-naphthyl) ethylene diamine. A 96-well plate containing equal volumes of sample and Griess reagents was incubated for 15 min at room temperature, covered from the light. A microplate reader was used to detect absorbance at 540 nm. A standard curve was generated using serial dilutions of sodium nitrite provided in the kit, and NO levels in samples were calculated from the standard curve and expressed as µmol/L. The absorbance from blank wells was subtracted prior to data analysis.

#### Measurement of *NF-κB Bcl-2, Bax, HO-1, and Nrf2*

The quantitative analysis of the serum inflammatory marker *NF-κB* was identified in vitro using the Human *NF-κB* ELISA kit (Catalog No. MBS450580). In accordance with the manufacturer’s guidelines, all reagents and standards in the kit were prepared. Spectrophotometric analysis was performed at 450 nm to assess the color variation. Additionally, the B-Cell Leukemia/Lymphoma 2 (*Bcl*-*2*) was detected using Cloud-Clone Corp, USA, Catalog No. SEA778Hu while, the proapoptotic marker (*BAX*) was detected using Cloud-Clone Corp, USA, Catalog No. SEB343Hu, Human *HO*-*1*(Heme Oxygenase 1) ELISA Kit (Elabscience, USA, **Cat**. **No**: E-EL-H2172) was used for HO-1 ELISA assay. Human Nuclear Factor Erythroid 2 Related Factor 2 (NFE2R2) ELISA kit **Cat**. **No**: MBS744368 was used for *Nrf2* evaluation. In accordance with the manufacturer’s guidelines, all reagents and standards in the kit were prepared.

### In-vivo experiment

#### Ethical approval

This study is conducted in accordance with ARRIVE guidelines^32^. All procedures in this experiment were approved and performed in accordance with guidelines and regulations of the animal ethical committee of the Uresearch Animal Facility-Institutional Animal Care and Use Committee (URAF-ICUAC) that organized and operating according to Cairo University, Institutional animal care and Use Committee CU-IACUC, Egypt. (Approval number: URAF-F-7-25).

#### Quarantine period

The National Research Center’s (NRC) animal housing unit supplied 18 healthy adult female albino rats weighing between 170 and 200 g, aged between 2 and 2.5 months. All animals were subjected to a mandatory quarantine period upon arrival at the facility in order to protect their well-being and health.

The rats were housed in controlled lighting, temperature, and humidity settings, with unrestricted access to food and water, while being kept clean. All experimental procedures were handled in compliance with the guiding standards.

#### Excisional wound creation

After the acclimatization period, the experimental rats were anesthetized intraperitoneally (IP) by a combination of Ketamine HCl (80 mg/kg) / Xylazine HCl (8 mg/kg)^30^. The skin hair on the dorsum (thoraco-lumber) was removed with an electric clipper and the site was cleaned with 70% alcohol followed by aseptic creation of 2 cm full-thickness skin excisions in each rat with the aid of scissor, scalpel, and marker.

#### Grouping

18 adult female healthy rats were then assigned randomly to three groups of equal numbers (*n* = 6) as follows:

- Group 1 (negative Control): wounded rats without any treatment.
- Group 2 (Positive Control): wounded rats were treated topically with pure NEG consisting of tea tree oil and jojoba oil as a nanoemulgel formula without propolis.
- Group 3 (Propolis NEG group): wounded rats were treated topically with Propolis NEG, daily for 2 weeks.

To avoid allocation bias in this study, animals were randomly assigned to the experimental groups following wound induction using a simple randomization procedure. Each rat was given an identification code, and group assignment was carried out separately from the investigators responsible for outcome assessment.

#### Estimation of wound closure percent (WC %)

The wound diameter was measured using a digital caliper for the calculation of wound closure percent (WC %) for each group at day 0, 3,7, and 14 from the following Eqs.^41,42^.

3 $$\:{\%}\:\mathrm{W}\mathrm{o}\mathrm{u}\mathrm{n}\mathrm{d}\:\mathrm{c}\mathrm{l}\mathrm{o}\mathrm{s}\mathrm{u}\mathrm{r}\mathrm{e}\:=\:\left.\left(\:\frac{wound\:area\:at\:day\:of\:wounding-wound\:area\:on\:day\:n}{wound\:area\:at\:day\:of\:wounding}\right.\right)\times\:100$$

Where n = number of days (7, 14) where the healed area was read.

#### Tissue sampling

On the 7th, and 14th days of the experiment, rats from every group were euthanasia with an overdose of Ketamine HCl/ Xylazine HCl^30^. The wound tissues of 7th days was being fixed in 10% neutral buffered formalin (NBF) for examination of histopathology. The tissues from the 14th day of the wounds were immediately split into two sections; the first section was used for histopathology, and the second portion was immersed in phosphate buffered saline (PBS) to homogenize it.

#### Tissue homogenization

The full cutaneous wound tissue was perfused with PBS (pH 7.4) and centrifuged at 4000 rpm (4 °C) for 15 min to collect the supernatant.

#### Antioxidant and lipid peroxidation

Superoxide dismutase (SOD) and malondialdehyde (MDA) levels was evaluated in the homogenized wound tissues according to the manufacturer’s instructions using kits from Biodiagnostic, Cairo, Egypt.

#### Determination of TNF- α

ELISA kits (Cloud Clone Corp Company, Houston, TX, USA) was used to measure the amount of tumor TNF-α in wound tissue homogenate in accordance with the manufacturer’s instructions.

#### Histopathological examination

I. ***Hematoxylin and eosin staining***.

After being fixed in 10% NBF, the tissue specimens were dehydrated in different alcohol concentrations (70, 80, 90, and 100) before being cleared in xylene and embedded in paraffin wax for histological sections. Using a Leica RM2235 rotating microtome, 5 μm slices were cut and stained with hematoxylin and eosin (H&E). Using a Leica light microscope (Leica Microsystems) coupled to a Leica ICC50 HD camera to capture digital images, the generated slices were investigated.

II. ***Masson’s trichrome staining***.

The specimens of wound tissue were stained with Masson’s trichrome (Sigma, USA) to examine the collagen, which was then stained green. The specimens were then examined under an Olympus CX43 light microscope that was connected to an Olympus DP27 camera to obtain digital images using CellSens dimensions software (Olympus).

### Statistical analysis

The obtained results were expressed as mean ± SD which were evaluated by One-Way Analysis of Variance (ANOVA) and followed by the Tukey test for multiple comparisons using Graph Pad Prism (version 8.0; Graph Pad Software Inc. San Diego, California, USA). For statistical significance, values of *p* < 0.05 were considered.

## Results

### GC-mass analysis

**Fig. 1:**
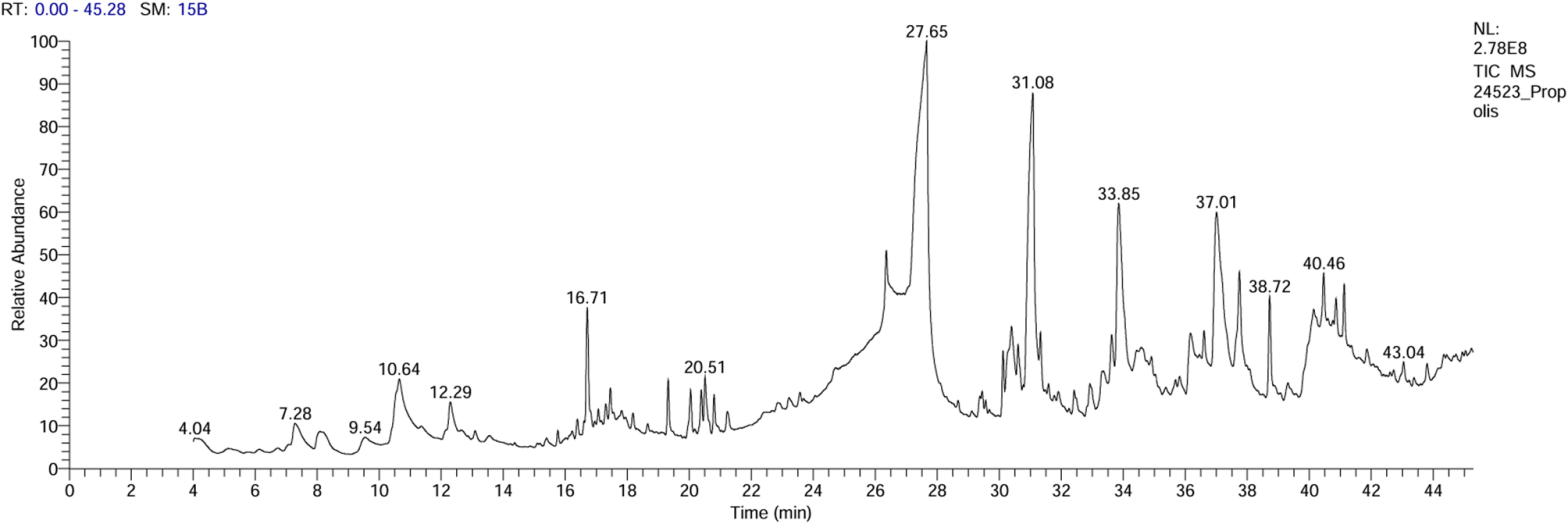
the GC-Mass spectrum of extracted propolis.

Figure 1’s GC-MS chromatogram of extracted propolis shows a total of many peaks that belong to bioactive chemicals that were found by comparing their mass spectrum fragmentation patterns to those of recognized compounds listed in NIST14 and WILEY 09 databases. Based on their molecular formula, peak area (%), and retention duration, the discovered chemical constituents are listed in Table 1. As shown, the major components are 1-(1,5-Dimethyl-4-Hexenyl)-4-Methyl, 5-hydroxy-7-methoxyflavanone, 4-H-1-Benzopyran-4-one, 5-hydroxy-7-methoxy-2-phenyl, hexadecanoic acid, octadecanoic acid, and benzoene ethanol.

**Table 1:** Chemical composition of extracted propolis via GC-Mass analysis, Retention time (RT), area %, MF, and molecular formula of each compound.

No.	RT	Compound	Area %	MF	Molecular formula	CAS. No.	Library
1	7.28	Benzene ethanol	0.86	779	C8H10O	60-12-8	WileyRegi stry8e
2	8.06	2,3-dihydro-3,5-dihydroxy-6-methyl-4 h-pyran-4-one	1.48	848	C6H8O4	28564-8 3 − 2	WileyRegi stry8e
3	9.54	6-o-hexopyranosylhexose #	0.68	594	C12H22O11	585-99-9	WileyRegi stry8e
4	10.64	Acetamide, 2,2,2-trifluoro-n-(2-phenyl ethyl)	1.21	689	C10H10F3NO	458-85-5	WileyRegi stry8e
5	12.29	Phenol, 2-(1,1-dimethylethyl)	0.84	792	C10H14O	88-18-6	WileyRegi stry8e
6	16.71	Benzene, 1-(1,5-dimethyl-4-hexenyl)-4-methyl	3.04	908	C15H22	644-30-4	WileyRegi stry8e
7	20.51	2-(4a,8-dimethyl-1,2,3,4,4a,5,6 ,8a-octahydro-2-naphthale nyl)-2-propanol	1.33	904	C15H26O	473-16-5	WileyRegi stry8e
8	27.65	Hexadecanoic acid	23.70	913	C16H32O2	57-10-3	WileyRegi stry8e
9	31.08	Octadecanoic acid	13.38	908	C18H36O2	57-11-4	Mainlib
10	33.85	5-hydroxy-7-methoxyflavanone	5.78	900	C16H14O4	75291-7 4–6	Mainlib
11	37.01	4 H-1-Benzopyran-4-one, 5-hydroxy-7-methoxy-2-phenyl	7.79	860	C16H12O4	520-28-5	Replib
12	38.72	Phenethyl palmitate	2.33	894	C24H40O2	72934-1 2–4	Mainlib
13	40.64	1,2-Propanediol, 3-(octadecyloxy)-, diacetate	1.23	649	C25H48O5	21994-8 1 − 0	Mainlib
14	43.04	9-octadecenoic acid, (2-phenyl-1,3-dioxolan-4-yl) methyl ester, cis	0.60	780	C28H44O4	56599-4 5 − 2	WileyRegi stry8e

### Physico-chemical characterization

**Fig. 2:**
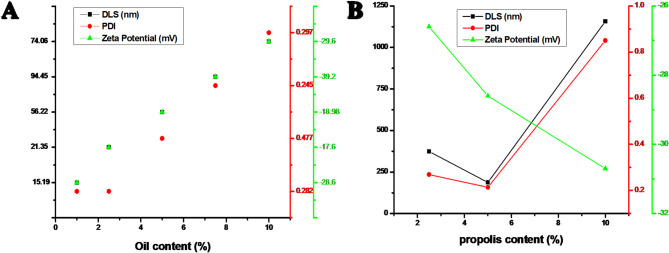
**A)** The effect of oil content and **B**) The propolis to oil content on zeta potential, DLS, and PDI of as-prepared formulations respectively.

Based on our previous findings^30^, the optimal ratio of jojoba oil to tea tree oil (TTO) was established as 2:1 and was therefore maintained in the present study. This ratio provided the most effective balance between emulsification efficiency and formulation stability. The droplet size, polydispersity index (PDI), and zeta potential of the NE formulations (FN1–FN5) showed significant changes. The particle size gradually increased from 15.19 ± 1.04 nm (FN1) to 94.45 ± 1.47 nm (FN4), as illustrated in Table 2; Fig. 2A. For FN5, the particle size slightly decreased (74.06 ± 1.69 nm). Because of adequate surfactant coverage and reduced coalescence, the smaller droplet sizes seen in FN1 and FN2 (15.19–21.35 nm) indicate effective emulsification at lower oil concentrations (1–2.5%). In contrast, the larger particle size of FN4 (94.45 nm) indicates droplet aggregation at higher oil concentrations (7.5%), whereas the slight reduction in FN5 (10%) may reflect surfactant saturation effects^43,44^. The majority of formulations exhibited uniform to moderate droplet distributions, according to PDI values ranging from 0.245 ± 0.016 (FN4) to 0.477 ± 0.11 (FN3). However, FN3 had more variability, which could have been attributed to partial surfactant insufficiency at the intermediate oil content (5%)^45–47^. Zeta potential values ranged from − 18 to − 39.2 mV. FN4 showed the highest magnitude (–39.2 mV), indicating significant electrostatic repulsion and improved colloidal stability^48^. Because it combines ideal zeta potential, structural integrity appropriate for long-term applications, and optimal droplet uniformity, FN4 can thus be regarded as the most stable NE.

As shown in Fig. 2B, the corresponding particle size, polydispersity index (PDI), and zeta potential were evaluated after adding various amounts of propolis extract (2.5%, 5%, and 10%) to the optimized NE system. The results revealed an evident concentration-dependent tendency in droplet size. Obviously, the mean particle size increased upon increasing propolis content, with 374.2 ± 4.89 nm for F-PN1, 187.8 ± 3.29 nm for F-PN2, and 1157 ± 87.41 nm for F-PN3. F-PN2 displayed a significantly smaller droplet size than F-PN1 due to enhanced interfacial stability and optimal interactions between the propolis extract and surfactant molecules, which decreased droplet coalescence. By contrast, the significant increase in droplet size for F-PN3 can be attributed to the high viscosity that prevents effective emulsification and aggregation caused by inadequate surfactant coverage of the larger organic phase^45^.

**Table 2:** Composition of different formulations and characterization of nano-emulsion based on particle size, polydispersity index (PDI), and zeta potential.

Formula	Propolis extract	Oil content	Span 80 (W/W %)	Tween 80 (W/W %)	SLS (W/W %)	Lecithin (W/W %)	DDH_2_O	DLS (nm)	PDI	Zeta Potential (mV)
**FN1**	0	1%	0.975%	6.525%	0.005%	0.05%	Q.s. to 100%	15.19 ± 1.04	0.282 ± 0.059	-28.6 ± 5.85
**FN2**	0	2.5%	0.975%	6.525%	0.005%	0.05%	Q.s. to 100%	21.35 ± 1.36	0.282 ± 0.117	-17.6 ± 1.26
**FN3**	0	5%	0.975%	6.525%	0.005%	0.05%	Q.s. to 100%	56.22 ± 1.10	0.477 ± 0.11	-18.98 ± 1.93
**FN4**	0	7.5%	0.975%	6.525%	0.005%	0.05%	Q.s. to 100%	94.45 ± 1.47	0.245 ± 0.016	-39.2 ± 2.1
**FN5**	0	10%	0.975%	6.525%	0.005%	0.05%	Q.s. to 100%	74.06 ± 1.69	0.297 ± 0.064	-29.6 ± 0.30
**F-PN1**	2.5 of (7.5)	7.5%	0.975%	6.525%	0.005%	0.05%	Q.s. to 100%	374.2 ± 4.89	0.269 ± 0.004	-26.6 ± 0.76
**F-PN2**	5 of (7.5)	7.5%	0.975%	6.525%	0.005%	0.05%	Q.s. to 100%	187.8 ± 3.29	0.214 ± 0.019	-28.6 ± 1.91
**F-PN3**	10 of (7.5)	7.5%	0.975%	6.525%	0.005%	0.05%	Q.s. to 100%	1157 ± 87.41	0.85 ± 0.016	-30.7 ± 0.513

**Fig. 3:**
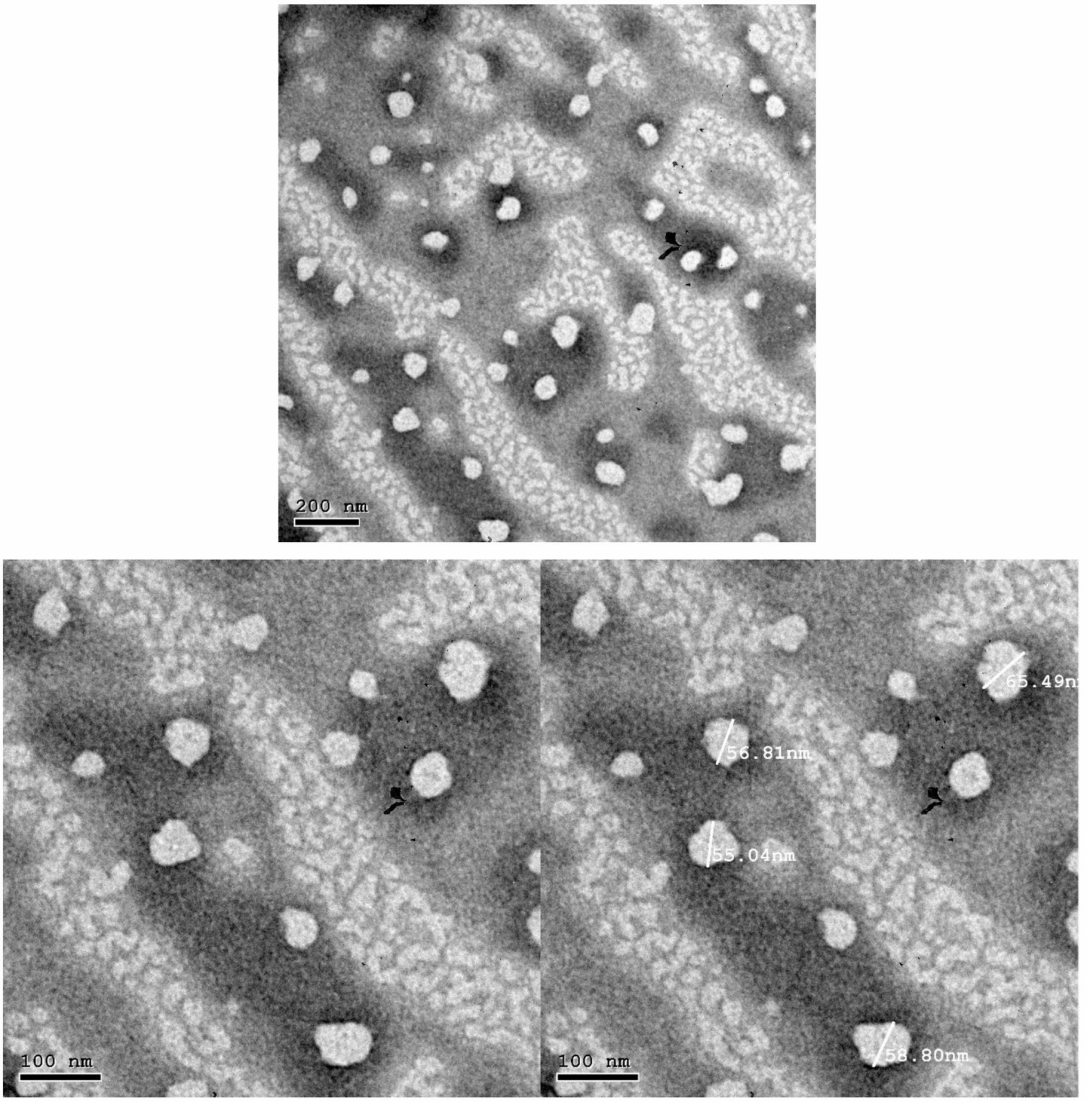
TEM images of optimized Nano-emulsion (FNP2).

To investigate the optimized nanoemulsion’s (F-PN2) particle size and surface morphology, Transmission Electron Microscopy (TEM) investigation was performed. As observed in Fig. 3, the TEM micrographs showed spherical droplets that were evenly distributed and had typical sizes of 18 to 34 nm. This morphological result confirms the formulation’s homogeneity, as provides the low PDI value (0.245).

### In-vitro release, Ex-Vivo permeation and skin retention

**Fig. 4:**
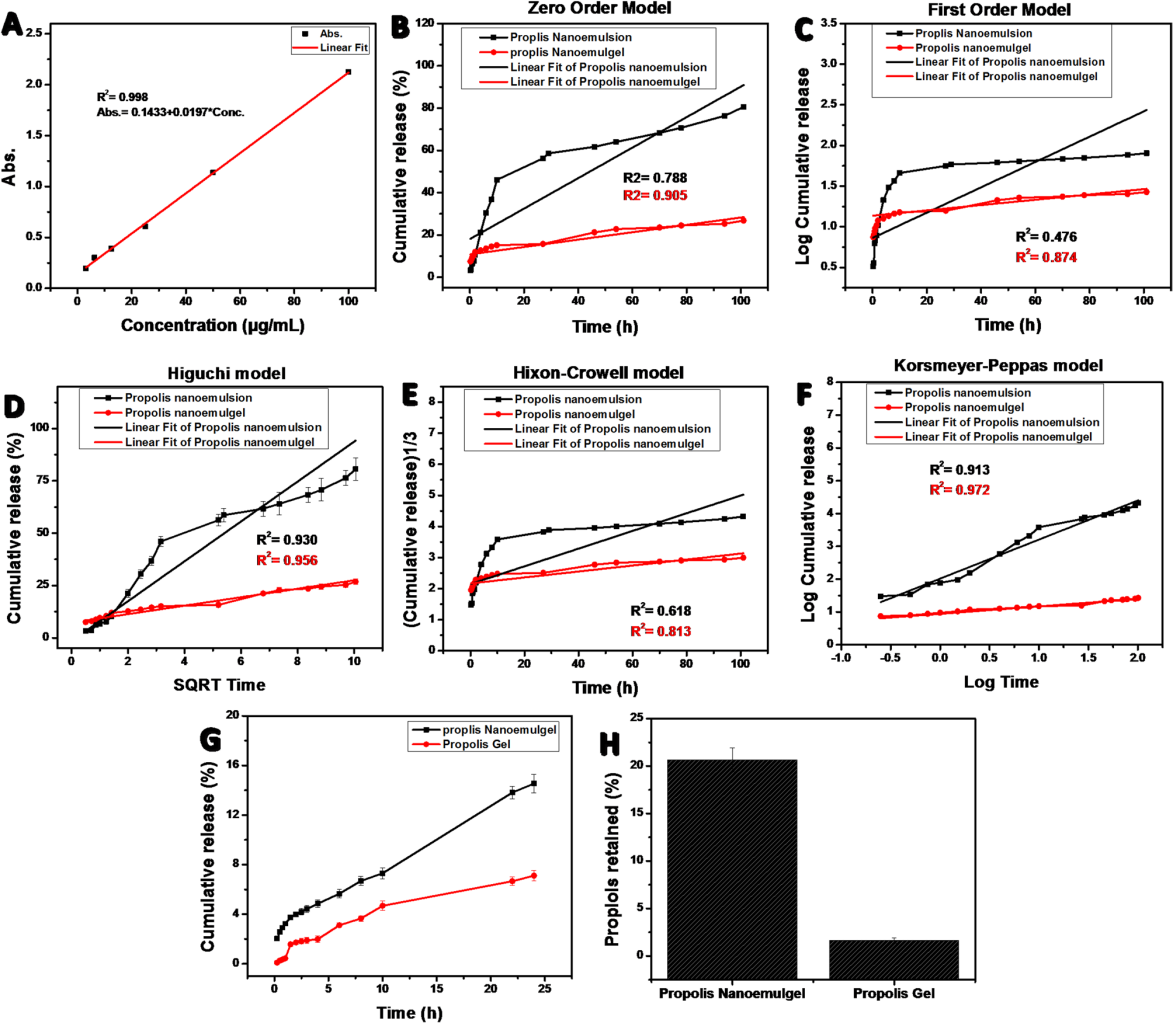
**A**) Calibration curve of propolis extract in PBS pH 7.4 contains tween-80 (1% w/w) at 265.2 nm, (B–F) *in vitro release kinetics of propolis from* NE *and* NEG. Kinetic models of cumulative release: (**B**) Zero-order, (**C**) First-order, (**D**) Higuchi, (**E**) Hixson–Crowell, and (**F**) Korsmeyer–Peppas models, **G**) the skin permeation of Propolis from propolis Extract gel and Propolis NEG, and **H**) The amount of Propolis Extract gel and Propolis NEG (F-PN2) retained in the skin after 24 h.

Figure 4**(A-F).** illustrate the Calibration curve of Propolis extract in PBS pH 7.4 contains tween-80 (1% w/w) at 265.2 nm, *and* Kinetic models of cumulative release: Zero-order, First-order, Higuchi, Hixson–Crowell, and Korsmeyer–Peppas models, respectively.

The release kinetics results suggest that propolis release from both the NE and NEG formulations is primarily governed by a diffusion-controlled mechanism, with additional contributions from polymer relaxation and matrix swelling. The high correlation with the Korsmeyer–Peppas model (R² = 0.913 and 0.972) indicates a non-Fickian (anomalous) transport behavior, where both diffusions of propolis molecules through the carrier and structural relaxation of the matrix influence the release rate.

The ex-vivo skin permeation and retention results **(**Fig. 4**(G–H))** revealed that the Propolis NEG exhibited superior dermal localization compared to the propolis extract Gel, with approximately 14.58% permeation and 20.64% skin retention after 24 h. Propolis gel, on the other hand, displayed much lower levels of 1.625% and 7.11%, respectively. The NE -based material is responsible for this enhanced effectiveness since the smaller droplets greatly increase the surface area and allow for closer interaction with the stratum corneum, which improves penetration. Furthermore, the NEG ‘s Carbopol 940 polymeric matrix promotes in occlusive film development and prolonged drug release, both of which improve hydration and consequently, skin permeability. Other NEG systems using natural bioactive have shown similar results, with nanoscale dispersion improving localized therapeutic effect and drug retention^49^. This study’s higher skin retention indicates that the NEG promotes localized delivery over systemic diffusion, reducing the possibility of adverse effects while preserving therapeutic levels at the wound site. A promising platform for the regulated dermal administration of natural chemicals in the treatment of chronic wounds and other topical applications is thus presented by the Propolis NEG.

### Biological assays

**Fig. 5:**
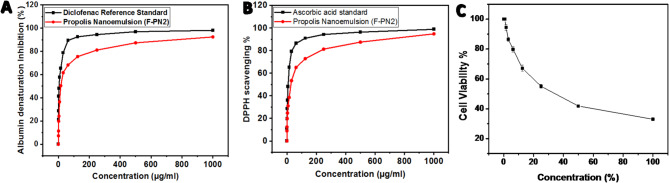
**A**) Albumin denaturation inhibition (%) for the propolis NE and diclofenac reference standard, **B**) The antioxidant activity of ascorbic acid (control) and propolis NE, and **C**) In-vitro cytotoxicity assay of the propolis NE on HSF-1cell line by MTT assay.

Figure 5A presents the albumin denaturation inhibition profile, indicating the anti-inflammatory potential of propolis NE (F-PN2) compared to Diclofenac. F-PN2 exhibited a concentration-dependent inhibition, achieving a maximum of 92.34% at 1000 µg/mL and maintaining activity (7.25%) at 0.5 µg/mL with IC_50_ = 15.45 ± 0.61 µg/ml.

Figure 5B illustrates the DPPH free radical scavenging activity of propolis NE (F-PN2) compared to ascorbic acid. F-PN2 exhibited strong antioxidant activity in a concentration-dependent manner, reaching a maximum of 94.83% at 1000 µg/mL. Although slightly lower than ascorbic acid (98.83%). with IC_50_ = 27.61 ± 1.02 µg/ml.

Furthermore, as indicated in Figure 5C, the cytotoxic effect of NE against skin fibroblast skin cell line HSF-1cell line under experimental conditions was (CC_50_ = 24.82± 0.16%) The highest concentration tested (1000 µg/mL) resulted in approximately 94.08% nhibition of cell viability, reflecting marked cytotoxicity.

**Fig. 6:**
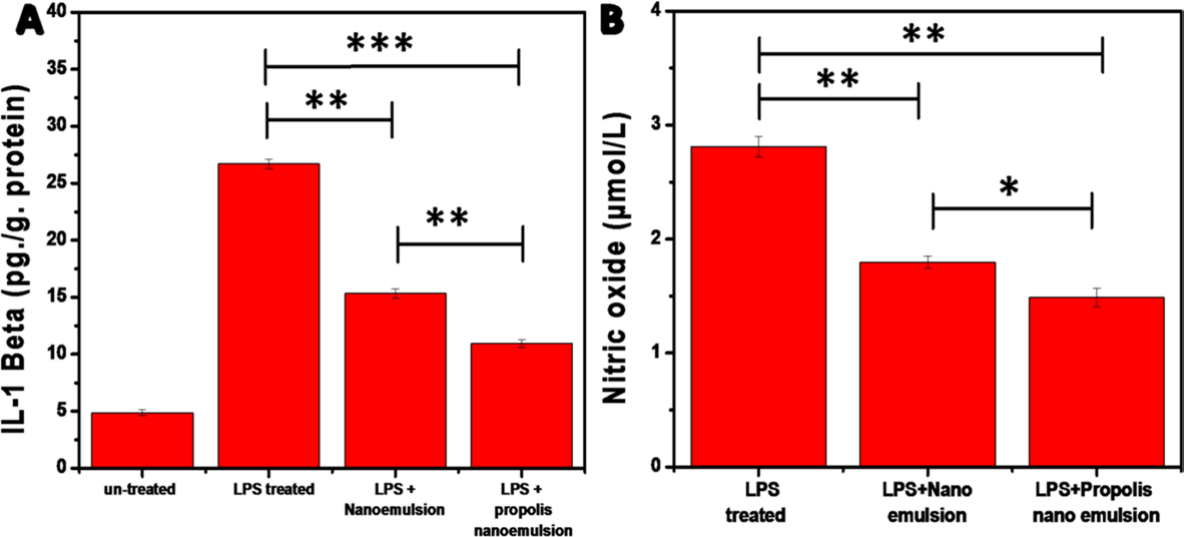
Effect of nanoemulsion and propolis nanoemulsion with (20% v/v) on *interleukin-1β (IL-1β)* (**A**) and Nitric oxide level (**B**) produced by LPS-stimulated macrophages.

The *IL-1β* (Fig. 6A) demonstrates a compelling dose-response-like suppression of LPS-induced inflammation in NHDF-Ad cells, with propolis nanoemulsion outperforming both untreated LPS control and blank nanoemulsion. The ~ 42% reduction (~ 26 to ~ 15 pg/mL) with propolis nanoemulsion could be assigned to enhanced penetration and bioactive delivery propolis and nanoemulsion’s lipophilic carrier improving cellular uptake over blank nanoemulsion.

Overall, the propolis nanoemulsion’s significant suppression of *IL-1β* suggests that it has considerable value as an anti-inflammatory nano-therapeutic, especially in cases where cytokine production is excessive. This result provides biological justification for its further investigation in models of inflammatory diseases and wound healing.

The results presented in Fig. 6B demonstrate a marked variation in nitric oxide (NO) levels among NHDF-Ad cells subjected to different treatments, as reflected by NO concentrations (µmol/L). LPS-treated NHDF-Ad cells exhibited the highest nitric oxide level (2.81 ± 0.088 µmol/L), indicating an elevated basal production of NO, which is commonly associated with oxidative stress and inflammatory responses in fibroblasts. Cells treated with nanoemulsion showed a reduction in nitric oxide levels (1.79 ± 0.055 µmol/L) compared to LPS-treated NHDF-Ad cells, indicating a moderate NO-suppressive effect, likely attributed to the known antioxidant constituents of jojoba and tea tree oil. Furthermore, propolis nanoemulsion treatment produced the lowest NO level of all investigated groups, with a significant decrease in nitric oxide production (1.48 ± 0.055 µmol/L). Propolis’s increased anti-inflammatory and antioxidant features when prepared as a nanoemulsion is highlighted by this notable reduction, which points to a robust inhibitory effect on nitric oxide production.

**Fig. 7:**
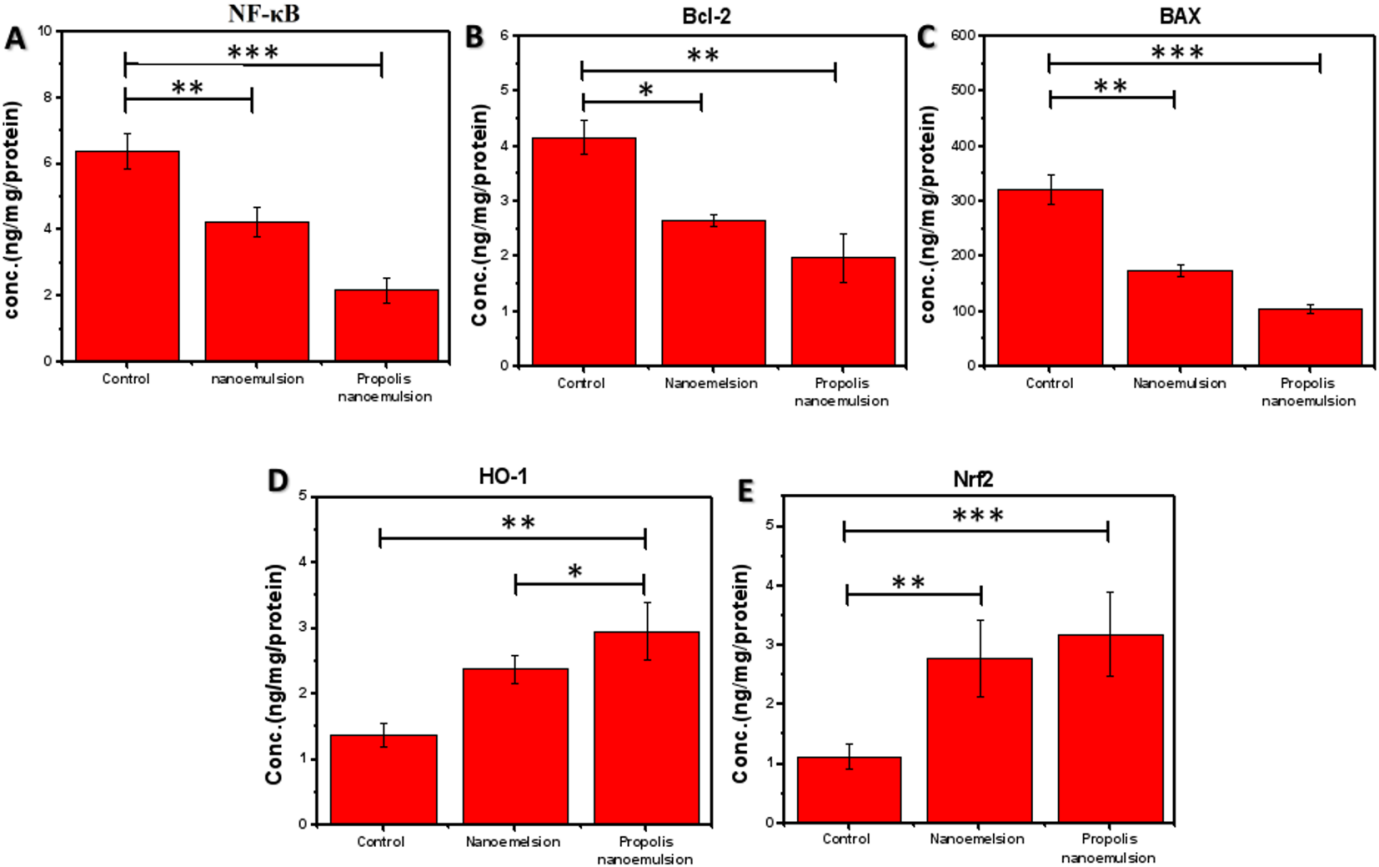
Effect of nanoemulsion and propolis nanoemulsion on *NF-κB*, *Bcl-2*, *BAX*, *HO-1*, and *Nrf2* expression levels in skin cells.

Figure 7 showed that there was a statistically significant difference between the groups studied for *NF-κB, Bcl-2, BAX, HO-1*, and *Nrf2* expression levels. As illustrated in Fig. 7A The control group exhibited the highest *NF-κB* expression level (6.39 ± 0.53 ng/ml), whereas treatment with nanoemulsion significantly decreased *NF-κB* to 4.25 ± 0.45 ng/ml. The propolis nanoemulsion group exhibited an additional significant reduction (2.1813 ± 0.3652 ng/ml), indicating highly significant differences in comparison to both the control and nanoemulsion groups (*p < 0.05, **p < 0.01, and ***p < 0.001). As shown in Fig. 7B, the anti-apoptotic protein (*Bcl-2*) displayed the highest expression level in the control group. Upon treatment with nanoemulsion *Bcl-2* level was reduced from 4.17 ± 0.31 ng/mg protein to 2.66 ± 0.98 ng/mg protein (*p < 0.05). while the significant reduction was reached after treatment with propolis nanoemulsion (1.98 ± 0.44 ng/mg protein, **p < 0.01). This decrease indicates that the treatments modify the signaling pathways that control cell survival and might impact the balance between cell survival and programmed cell death (apoptosis). In the same way, the control group had the most *BAX* expression (321.26 ± 27.25 ng/mg protein). The nanoemulsion treatment significantly reduced *BAX* levels to 174.89 ± 10.61 ng/mg protein. The propolis nanoemulsion group exhibited the lowest *BAX* level (105.31 ± 7.77 ng/mg protein), demonstrating a statistically significant reduction in comparison to both the control and nanoemulsion groups Fig. 7C. Together, the modification of *Bcl-2* and the downregulation of *BAX* suggest that nanoemulsion treatments, especially propolis nanoemulsion, have a major impact on apoptotic signaling pathways. Increased biological activity of the nano-formulated bioactive chemicals may contribute to better cellular protection and management of inflammatory or oxidative stress situations, as suggested by the greater suppression seen with propolis nanoemulsion. Additionally, *HO-1* expression levels (Fig. 7D) were significantly enhanced after treatment. Briefly, the control group showed the lowest level (1.38 ± 0.18 ng), while the treatment with nanoemulsion was enhance the level to 2.38 ± 0.22 ng. furthermore, the propolis nanoemulsion group exhibited the highest *HO-1* expression (2.96 ± 0.43 ng, *p* < 0.01), suggesting enhanced activation of antioxidant defense mechanisms. Similarly, *Nrf2* expression was significantly upregulated by both treatments (Fig. 7E**)**. Compared with the control group (1.12 ± 0.22 ng), nanoemulsion treatment significantly increased *Nrf2* levels to 2.77 ± 0.64 ng (*p* < 0.01), while propolis nanoemulsion produced the highest Nrf2 level (3.18 ± 0.71 ng, *p* < 0.001). This indicates a strong activation of the *Nrf2* **-**mediated antioxidant signaling pathway. Collectively, these findings demonstrate that nanoemulsion formulations, particularly propolis nanoemulsion, significantly activate the *Nrf2***/***HO-1* antioxidant pathway while modulating *BAX*/*Bcl-2* expression, suggesting a protective cellular response against oxidative stress.

### In-vivo study

#### Wound closure percent (WC %)

**Fig. 8:**
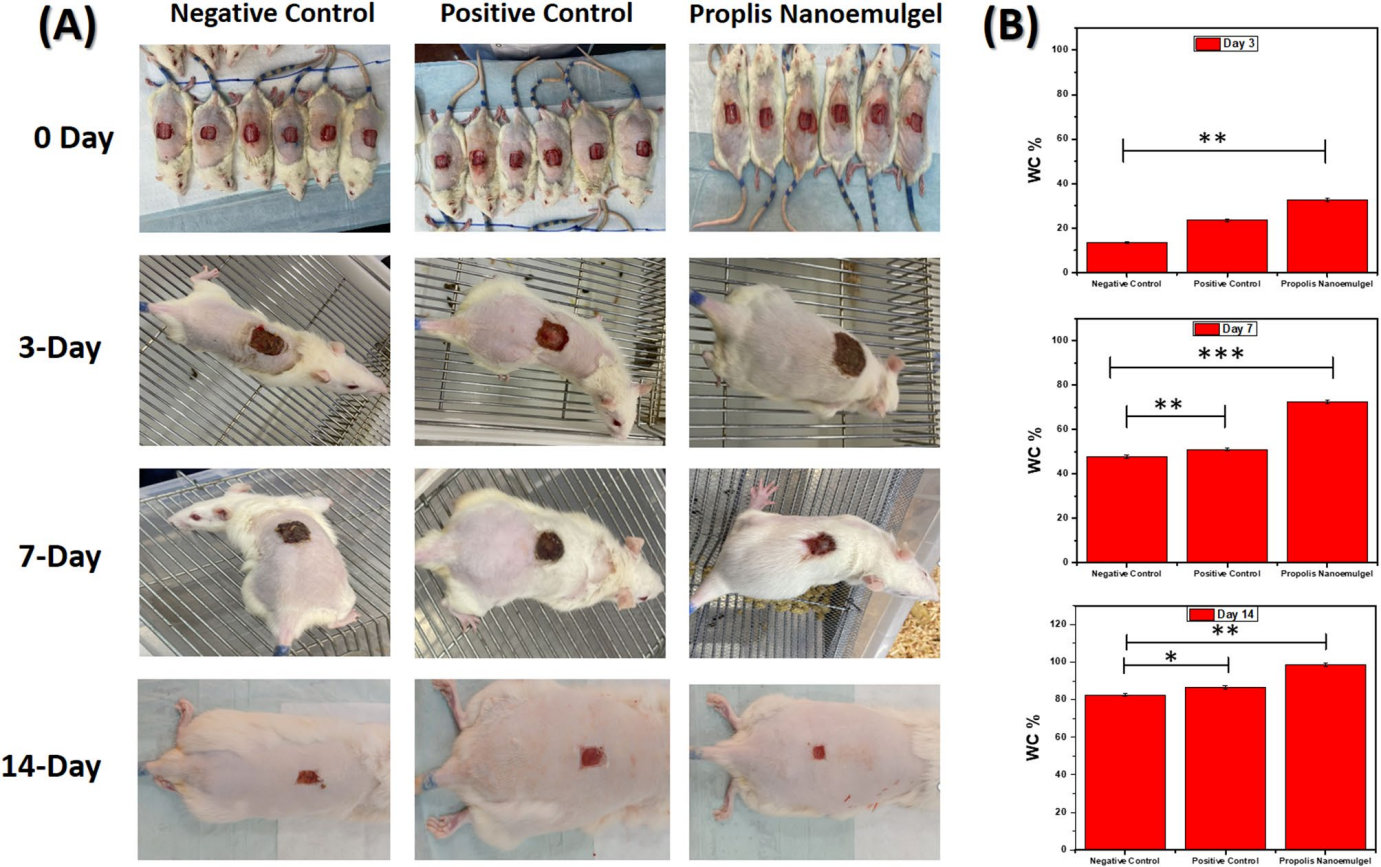
**A**) Images of the excisional skin lesions in various groups on days 0, 3, 7, and 14 following treatments and **B**) Mean *±* SD of WC% of negative control, positive control, and propolis NEG groups at days 3, 7, 14 post-treatments.

Photographic images on days 0, 3, 7, and 14 after excision wound induction was used to evaluate the effective in vivo WH response. Figure 8A shows representative views of the WH process in the propolis NEG -treated group (Group III), positive control (Group III), and negative control (Group I). As shown in Figs. 8B, the wound closure percentage (WC%) was assessed quantitatively. Day 3 showed a statistically significant increase in WC% in Group III over Group I (*p* < 0.01), indicating a considerable early constriction of the wound. In addition, Group III’s WC% increased noticeably although not significantly in comparison to Group II. By day seven, the WC% of Groups II and III was significantly higher than that of the Negative control (*p* < 0.01 and *p* < 0.001, respectively), with Group III achieving superior healing progress. On day 14, the propolis NEG-treated wounds (Group III) showed comparable or slightly superior efficacy to the positive control (Group II), reaching almost full closure with a statistically significant difference from the negative control (*p* < 0.01).

#### Antioxidant and lipid peroxidation in experimental rats

**Fig. 9:**
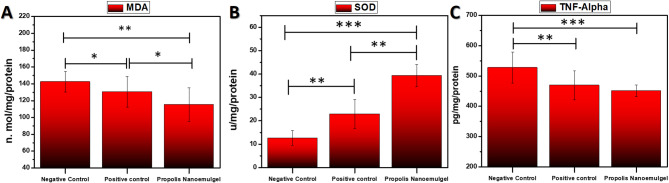
**(A**,** B)** MDA and SOD levels in healed tissue homogenates of cutaneous wounds for negative control, positive control, and propolis NEG groups albino rats at day 14 post-treatment. **(C)** TNF-α activities levels in homogenates of day-14 cutaneous wounds of negative control, positive control, and propolis NEG albino rats.

The oxidative stress markers shown in Fig. 9**(A-B)** show that the negative control group had noticeably higher MDA levels, which is an indication of lipid peroxidation. While both treatment groups displayed increased SOD activity and decreased MDA, the propolis NEG group’s antioxidant response was the most significant. Malondialdehyde (MDA) and superoxide dismutase (SOD) levels were measured in wound tissues of various groups to evaluate the oxidative stress parameters, as shown in Fig. 9**(A-B)** and SOD, respectively. Increased lipid peroxidation was indicated by the Negative control group’s notably greater MDA levels. MDA levels were significantly reduced by treatment with both the propolis NEG and the positive control, with the propolis-treated group showing the largest decrease, indicating improved resistance to oxidative stress. In contrast, SOD activity a key antioxidant defense enzyme was markedly increased in the wound tissues of both treated groups in comparison to the negative control. Notably, the propolis NEG group exhibited the highest SOD levels, indicating a potent antioxidant response.

#### Anti-inflammatory markers in rats᾿ wound tissues

Tumor necrosis factor-alpha (TNF-α), a pro-inflammatory cytokine, was significantly higher in the negative control group, shown in Fig. 9C, indicating a higher inflammatory response. On the other hand, TNF-α levels were considerably lower in the propolis NEG -treated group than in the positive control group, with the propolis-treated group showing the lowest concentration.

#### Histopathological examination

**Fig. 10:**
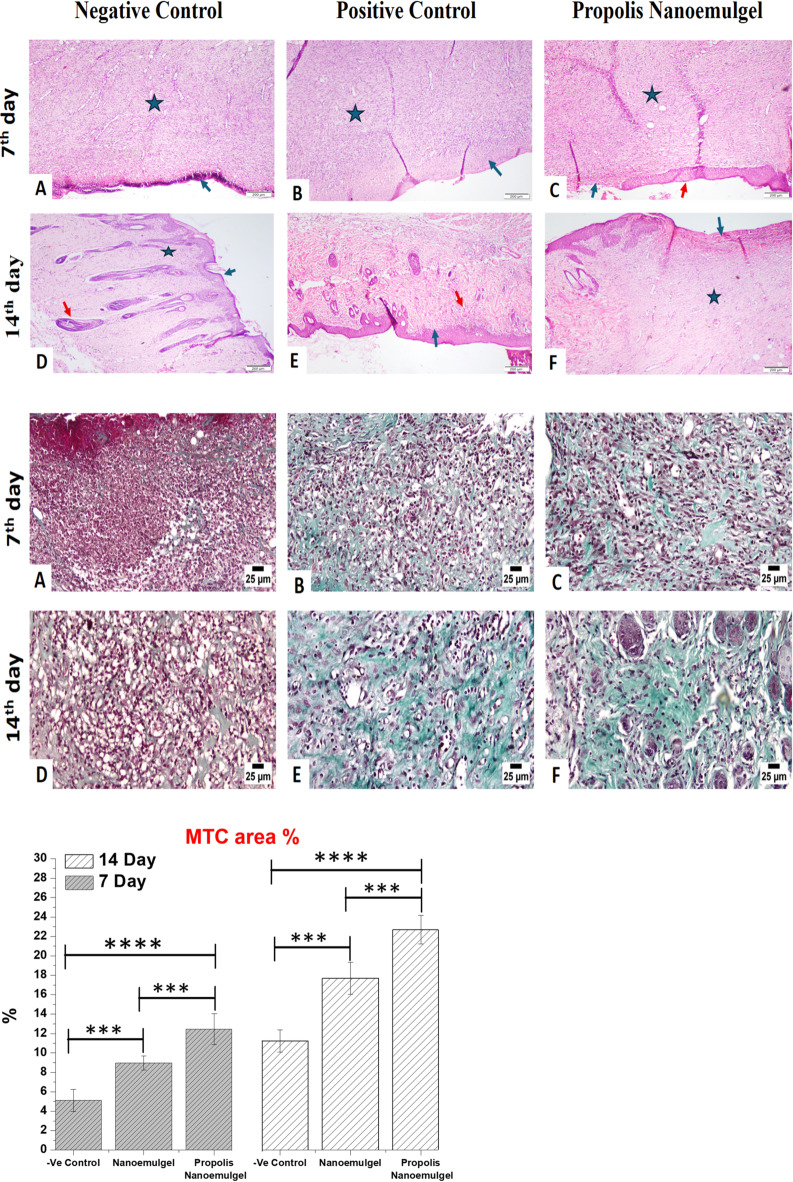
**H&E; on 7th day** (**A**), the wounds of negative control group shows superficial necrosis (blue-arrow) with polymorphnuclear leukocytic cells infiltration and underlying granulation tissue (star) ( X 40). (**B**), the wounds of NEG group showing superficial necrosis (blue-arrow) with polymorphnuclear leukocytic cells infiltration and underlying granulation tissue (star) (X 40). (**C**), skin of propolis NEG group showing superficial necrosis (arrow) with polymorphnuclear leukocytic cells infiltration and underlying granulation tissue (star). Reepithelization was observed at the edge of the wound (red arrow) (X 40). **On 14th day** (**D**), the wounds of negative control group showing fibrosis (star) with an overlying epidermis (arrow). note the presence of hair follicles (red arrow) (X 40). (**E**), the wounds of NEG group showing hyperplastic epidermis (arrow) and leukocytic cells infiltration in the dermis (red arrow) (X 40). (**F**), the wounds of propolis NEG shows superficial necrosis (arrow) w. **MTC; on 7th day** (**A**), the wounds of negative control show limited collagen bundles deposition in the wound gap. (**B**), the wounds of NEG group shows moderate collagen bundles deposition in the wound gap. (**C**), Photomicrograph of skin of propolis NEG group showing higher collagen bundles deposition in the wound gap. **On 14th day** (**A**), the wounds of negative control show lower collagen bundles deposition in the wound gap. (**B**), and (**C**), the wounds of NEG group and propolis NEG showing higher deposition of collagen bundles in the wound gap. **MTC area %;** On 7th and 14th days, values are presented as means ± SD. Significant difference is considered at P˂0.05.

Histological examination confirmed the macroscopic and biochemical results and showed that tissue regeneration varied significantly among the treatment groups. Animals from the negative control group (assessed at day 7 and day 14) displayed impair WH, reflected by the presence of sustained infiltration of inflammatory cells, destroyed dermal structure, and absence of re-epithelial layer (Fig. 10(A and D)). In contrast, the positive control had some improvement, with incomplete epithelialization and a decrease in inflammatory infiltration (Fig. 10B and E). Notably, propolis NEG treated wounds showed faster healing with early and complete re-epithelialization, organized collagen fiber deposition, and dermal annex structures regeneration at day 14 (Fig. 10(C and F)). These histological characteristics of the wound suggest a more mature stage of tissue remodeling with signs of increased fibroblast activity and limited extracellular matrix reconstruction^50,51^.

**Masson Trichrome (MTC).** Collagen deposition in negative control group was minimal at seven and fourteen days. Meanwhile, propolis NEG showed substantial collagen bundle deposition whereas, NEG group showed modest deposition (Fig. 10. MTC area Percent). The statistical analysis of the Masson’s trichrome (MTC) area % of collagen bundle deposition showed that Group 3 outperformed the other groups at 7 and 14 days.

## Discussion

Propolis extract, tea tree oil, and jojoba oil with well-reported biological activities were used to formulate and evaluate a propolis-based nanoemulgel (NEG) to achieve the delivery advantages, high thermodynamic stability, controlled drug release, and high biological activities. In the present study, the ethanolic maceration approach was used to extract the propolis from its ore and GC-Mass analysis was used to determine the its active constituents while, the low energy emulsification method was used to formulated a stable nanoformulation with tea tree and jojoba oil.

According to zeta potential tests, all formulations had negative surface charges between –26.6 mV (F-PN1) and –30.7 mV (F-PN3), which suggests sufficient electrostatic repulsion to prevent droplet aggregation and maintain colloidal stability. By integrating the smallest particle size, lowest PDI, and favorable zeta potential, F-PN2 (5% propolis) was found to be the most suitable formulation based on these physicochemical parameters, providing better stability and homogeneity^52^. Furthermore, the slight difference between the particle sizes determined by Dynamic Light Scattering (DLS) and TEM is explained by the nature of the observations; TEM provides the dry, hard-sphere diameter of solvated particles, while DLS gives the hydrodynamic diameter^30^. The nanosized droplets (<200 nm) observed in TEM further support the kinetic stability of the NE, minimizing gravitational separation, coalescence, and droplet aggregation^53^. These morphological findings further validated F-PN2 as the optimized NE, selected for subsequent physicochemical and biological evaluations.

The in vitro release kinetics of propolis NE and propolis NEG displayed the strong fit to the Higuchi model (R² = 0.930 and 0.956) further supports the dominance of diffusion as the main mechanism. Interestingly, the propolis NEG system exhibited higher correlation values across all models, particularly in the Korsmeyer–Peppas and Higuchi equations, indicating a slower and more controlled release pattern compared with the NE. This can be attributed to the presence of the Carbopol 940 polymeric network, which restricts molecular mobility, increases viscosity, and prolongs drug diffusion pathways^54,55^. In contrast, the NE allowed a faster initial release due to its lower viscosity and higher fluidity. The relatively poor fit to zero-order, first-order, and Hixson–Crowell models suggests that the release is not constant over time nor driven solely by dissolution or erosion processes. Additionally, the ex-vivo skin permeation and retention study demonstrate that, the propolis-loaded NE provides a sustained-release delivery system when it is embedded into a gel matrix. This improves the residence time and local bioavailability of propolis at the wound site, which is crucial for preserving sustained antioxidant and anti-inflammatory activity throughout the wound-healing process.

Subsequently, the calculated CC_50_ value was 24.82 ± 0.16%, representing the concentration at which 50% of cell viability was inhibited. This suggests that while the propolis NE exhibits cytotoxic effects at higher concentrations, it remains relatively safe at lower levels. These findings support the use of propolis NE at sub-cytotoxic concentrations such as 20% for further biological assays, including WH studies. After that, the propolis NEG-treated wounds (Group III) showed comparable or slightly superior efficacy to the positive control (Group II), reaching almost full closure with a statistically significant difference from the negative control (*p* < 0.01).

The DPPH assay and albumin denaturation assay demonstrated that, the developed propolis NE exhibited strong anti-inflammatory and antioxidant activities. Additionally, the nanoemulsion-based treatments significantly modulate key inflammatory, apoptotic, and antioxidant signaling pathways. As shown in Fig. 7A, *NF-κB* levels were markedly elevated in the control group, indicating activation of pro-inflammatory signaling. Treatment with nanoemulsion significantly reduced *NF-κB* expression, while propolis nanoemulsion produced a more pronounced suppression. Since *NF-κB* is a central regulator of inflammatory cytokine production and oxidative stress responses, its inhibition suggests that the treatments effectively attenuate inflammatory signaling. Additionally, the bioactive components of propolis, are known to inhibit the production of pro-inflammatory cytokines by blocking the *NF-κB* and *MAPK* signaling pathways. These performances are assigned to many factors. Firstly, the propolis’s composition of flavonoids and phenolic acids that reduce the inflammation by inhibiting the production of prostaglandins, cytokines, and inhibited inflammatory genes expression such as TNF-α and IL-6 in addition to suppress the NF-κβ activation and improve the antioxidant effect^56,57^. Furthermore, the synergetic effect of combination between propolis, Jojoba, and tea tree oil in which, the anti-inflammatory properties of tea tree oils are ascribed to the presence of terpinen-4-ol in the former and sterols and various tocopherols in the latter in addition to α, γ, and δ tocopherol in jojoba oil and which mean synergetic effect so this result show more significant enhancement of antioxidant activity than the previous studies^30,58,59^. This enhanced anti-inflammatory effect illustrates their synergistic effect.

The modulation of apoptosis-related proteins further supports this protective effect. *Bcl-2*, an anti-apoptotic marker, was significantly decreased following nanoemulsion treatment and further reduced in the propolis nanoemulsion group compared with the control group. In parallel, *BAX*, a key pro-apoptotic protein, showed a significant reduction after treatment, with nanoemulsion decreasing its expression and propolis nanoemulsion producing a stronger suppression. The coordinated modulation of *Bcl-2* and *BAX* suggests that the treatments regulate mitochondrial apoptotic signaling and may help maintain cellular homeostasis under inflammatory conditions. These significant decrease indicates that there is a significant anti-apoptotic activity through stabilize mitochondrial membranes, lower levels of reactive oxygen species (ROS), and block pro-apoptotic signaling pathways^60,61^. In addition to their anti-inflammatory and anti-apoptotic effects, the treatments also enhanced cellular antioxidant defense mechanisms, the expression of the antioxidant-related genes *HO-1* was significantly increased in the nanoemulsion group compared with the control, with a further elevation observed in the propolis nanoemulsion group. Similarly, *Nrf2* levels were markedly upregulated following treatment, showing significant increases in both the nanoemulsion and propolis nanoemulsion groups compared with the control. Since *Nrf2* is a key transcription factor that regulates antioxidant response elements and induces *HO-1* expression, the upregulation of the *Nrf2/HO-1* axis indicates activation of an important cytoprotective pathway^62,63^. The lack of *HO-1* may result in the formation of deep skin lesions and the total inhibition of re-epithelialization, as reported in earlier research, along with poor neovascularization. It’s interesting to note that propolis and its main components were also discovered to boost *HO-1* expression levels following UV irradiation at earlier time points, protecting immortalized human skin fibroblast cells (NB1-RGB) against UVR-induced cell death^64,65^. Thus, propolis’s ability to stimulate the expression of genes associated with antioxidants is likely correlated with its ability to heal wounds. Overall, these results suggest that synergetic capacity of nanoemulsion formulations (The propolis extract, TTO, and Jojoba oil) to regulate *NF-κB* signaling pathways, as well as activate, provides a mechanistic framework linking molecular pathways with systemic immune outcomes^65–67^.

The propolis nanoemulsion’s enhanced efficiency over the blank nanoemulsion illustrates the significance of incorporating active compounds rather than just the delivery method. These results demonstrate that; natural anti-inflammatory agents’ therapeutic efficacy can be enhanced by nanoemulsion-based delivery systems. Additionally, the enhancement in antioxidant properties of propolis NEG-treated group were characterized through lowering MDA level and improve the SOD in comparison with control groups. These findings are consistent with prior studies, which reported that topical application of essential oils as^68,69^. Furthermore, Abou-Zeid et al.^70^. demonstrated the efficacy of propolis and jojoba oil in lowering MDA and enhancing antioxidant enzyme activities such as SOD, GSH, and CAT in various tissues, supporting the current findings. The bioactive flavonoids and phenolic compounds of propolis NEG formula are known to change cellular responses by enhancing collagen deposition, angiogenesis, and fibroblast proliferation. This facilitates the healing cascade and accelerates the wound closure. Additionally, alter inflammatory pathways, as confirmed by lowering the TNF-α level. These findings are consistent with earlier research showing that propolis and tea tree oil significantly lowered TNF-α production in macrophage cells^71,72^. According to another studies, terpinen-4-ol, the main active ingredient in tea tree oil, inhibited lipopolysaccharide-stimulated monocytes from producing TNF-α^73,74^. Propolis NEG may have dual therapeutic benefits in promoting WH, as demonstrated by its anti-inflammatory properties and increased antioxidant activity. Collectively, the in vitro and in vivo results confirm that propolis nanoemulsion effectively modulates inflammatory, apoptotic, and antioxidant pathways, supporting its potential as a promising therapeutic strategy for enhancing tissue repair and wound healing.

Hence, the known pharmacological properties of propolis and the advantages of nano formulation due to better penetration of the drug into the skin lead to enhancement in wound healing. overall, propolis, tea tree oil, and jojoba oil NEG is a natural, bioactive, and promising topical formulation that has the potential to effectively replace traditional approaches for wound healing and infection prevention. Future studies will be focused on evaluating the formulation in models of chronic and infected wounds, as well as long-term safety and comparative efficacy assessments, to improve its clinical translation.

## Conclusion

A low energy homogenization method was used to successfully formulate propolis, tea tree oil, and jojoba oil into a stable nanoemulsion. The diffusion-controlled favorable drug release mechanism of the optimized formulation (F-PN2) has been demonstrated by the Korsmeyer–Peppas (R^2^ = 0.972) and Higuchi (R^2^ = 0.956) models. Significant anti-inflammatory effects (92.34% inhibition; IC_5_₀ = 15.45 µg/mL) and high antioxidant capacity (94.83% DPPH scavenging; IC_5_₀ = 27.61 µg/mL) due to synergetic effect between formula’s phenolic and flavonoid content. The propolis NE demonstrated superior dermatological performance than the propolis extract gel, with a much greater skin retention rate (~ 20.64%) and a higher skin permeation (~ 14.58%). Furthermore, the* IL-1β* and NO level demonstrate a compelling dose-response-like suppression of LPS-induced inflammation and oxidative stress in NHDF-Ad cells, with propolis nanoemulsion outperforming both untreated LPS control and blank nanoemulsion. The propolis nanoemulsion, significantly modulate key inflammatory, apoptotic, and antioxidant pathways. The observed downregulation of *NF-κB* and *BAX*, together with the activation of the *NRF2/HO-1* signaling pathway, indicates effective suppression of inflammation and oxidative stress while promoting cellular protection.WH in vivo study have demonstrated reduce in TNF-α expression, enhancement in superoxide dismutase (SOD) activity, and decreased malondialdehyde (MDA) levels, indicating improved oxidative stress balance and inflammatory control. Histopathological study verified complete re-epithelialization, well-formed granulation tissue, and accelerated healing. Propolis, tea tree oil, and jojoba oil all accelerated the healing process with their combination regenerative, anti-inflammatory, and antioxidant properties. In conclusion, propolis NEG is a natural, bioactive, and promising topical formulation that has the potential to successfully replace conventional methods for infection prevention and wound healing. This study has some limitations, so further future studies focus on assessing the formulation in models of infected and chronic wounds, as well as long-term safety and comparative efficacy evaluations to improve the clinical translation.

## Supplementary Information

Below is the link to the electronic supplementary material.

Supplementary Material 1

Supplementary Material 2

Supplementary Material 3

Supplementary Material 4

## Data Availability

All data generated or analyzed during this study are included in this published article, which are available from the corresponding author on reasonable request.
