# Supplementary material for: Development and evaluation of a propolis, tea tree oil, and jojoba oil nanoemulgel with enhanced antioxidant, anti-inflammatory, and wound-healing activities
Source: Sci Rep. 2026 May 25;16:16143. doi: 10.1038/s41598-026-50846-y (PMC13201651; doi:10.1038/s41598-026-50846-y)
Supplement: Supplementary file 3 — Supplementary Material 3 [file 41598_2026_50846_MOESM3_ESM.pptx]

## Slide 1
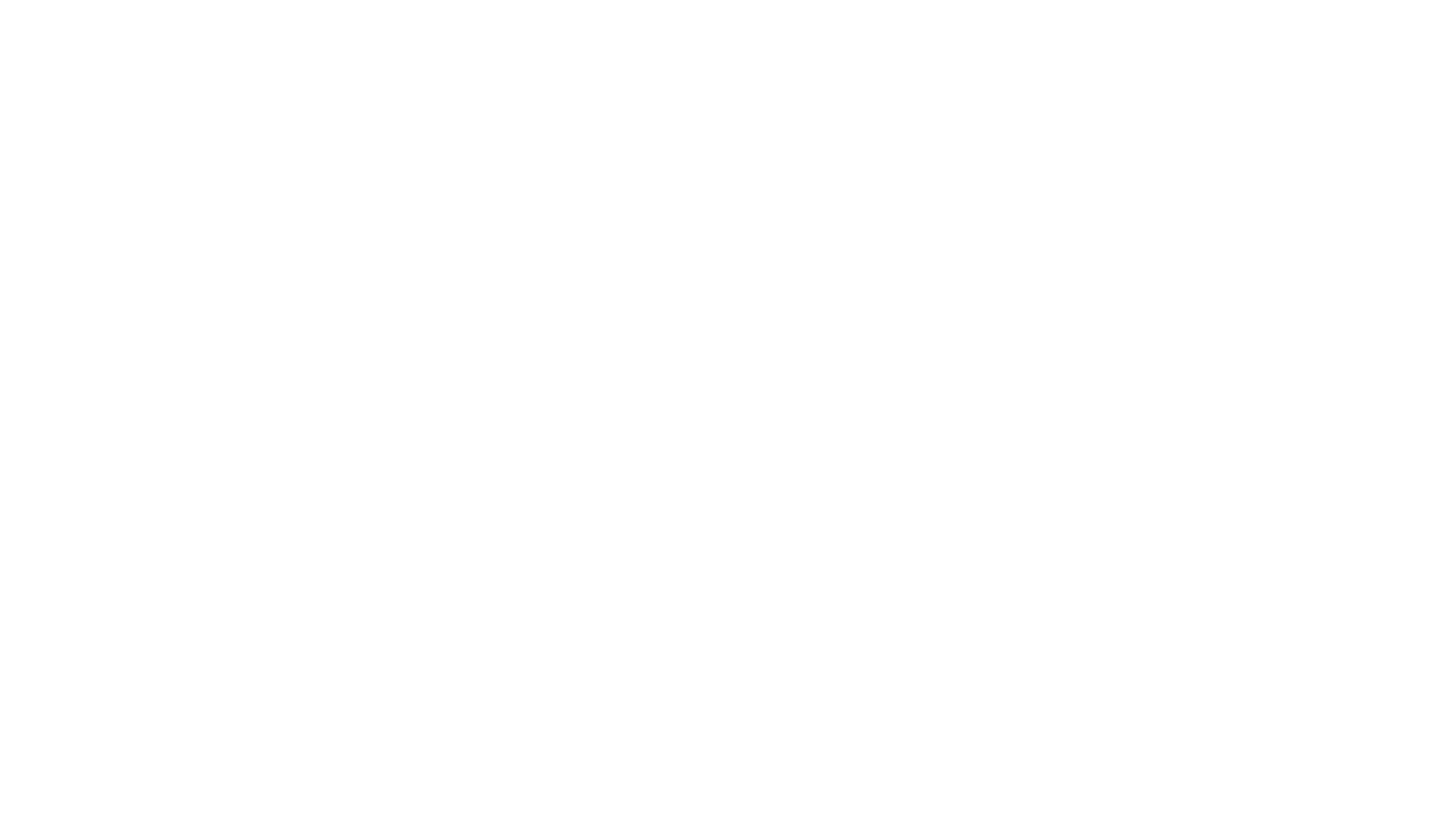

#

## Slide 2
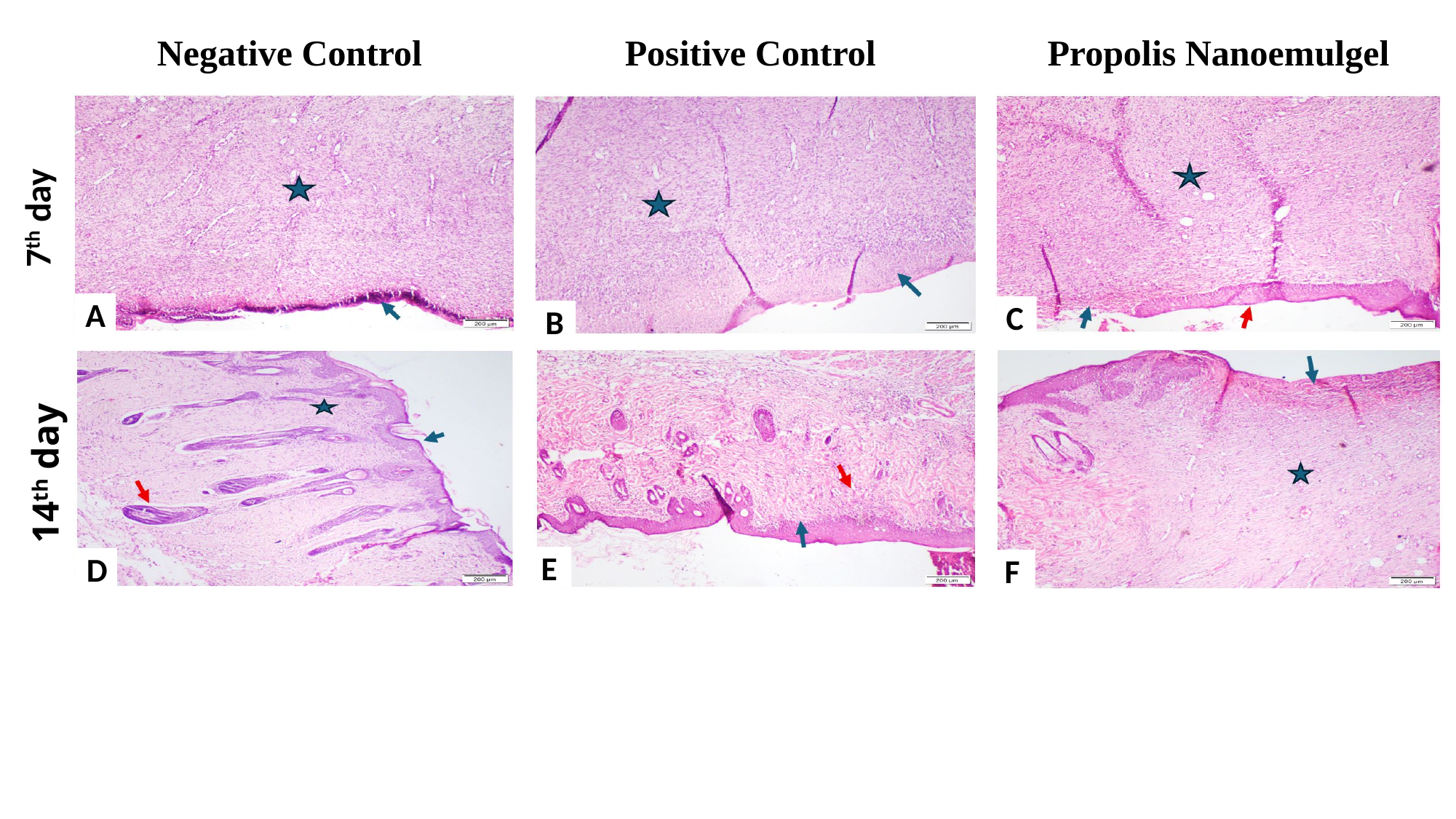

Positive Control
Propolis Nanoemulgel
Negative Control
7th day
A
C
B
14th day
E
D
F

## Slide 3
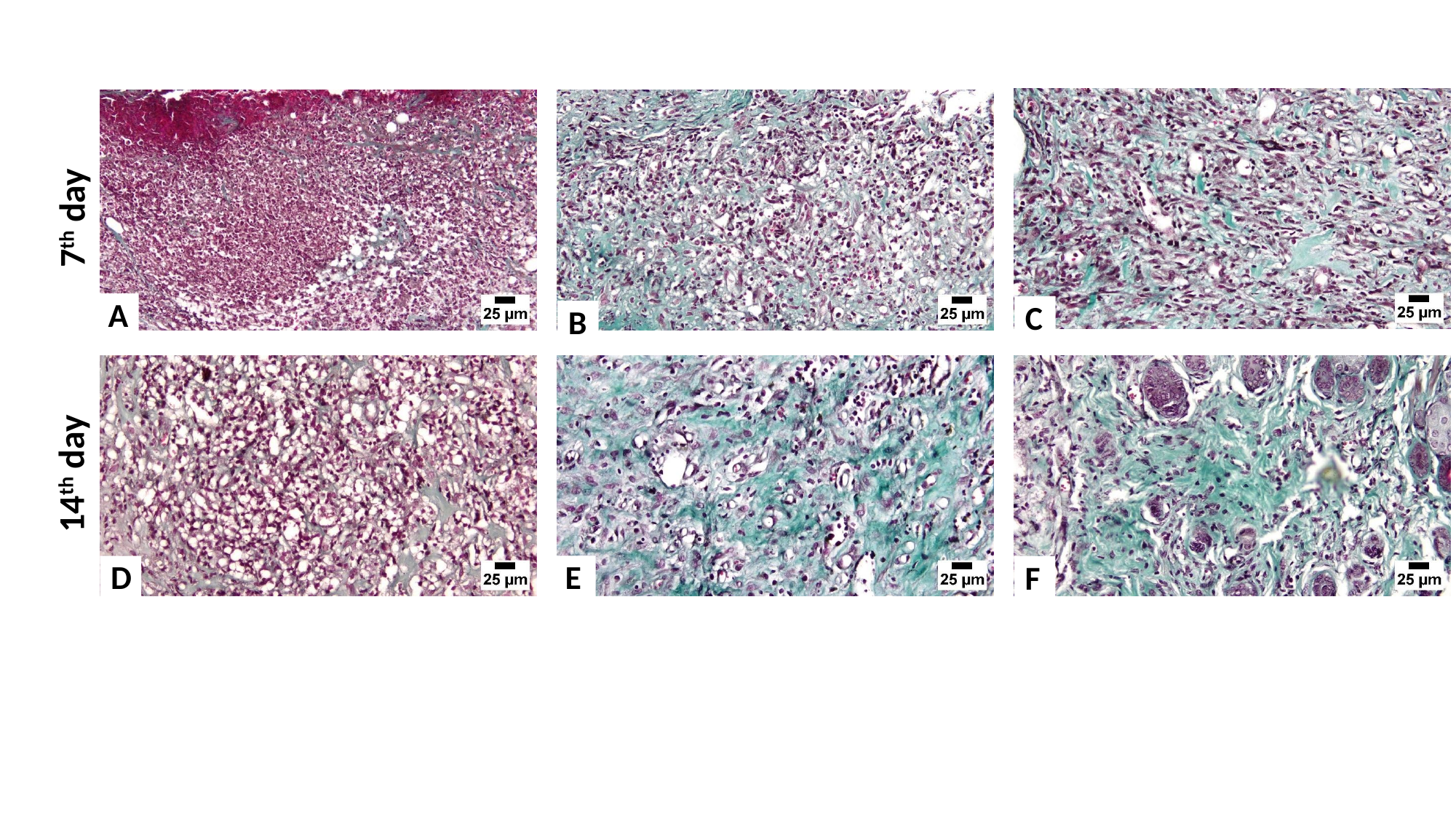

#
7th day
A
C
B
14th day
E
D
F
