# Supplementary figures and images for: Development and evaluation of a propolis, tea tree oil, and jojoba oil nanoemulgel with enhanced antioxidant, anti-inflammatory, and wound-healing activities
Source: Sci Rep. 2026 May 25;16:16143. doi: 10.1038/s41598-026-50846-y (PMC13201651; doi:10.1038/s41598-026-50846-y)

## Slide 1
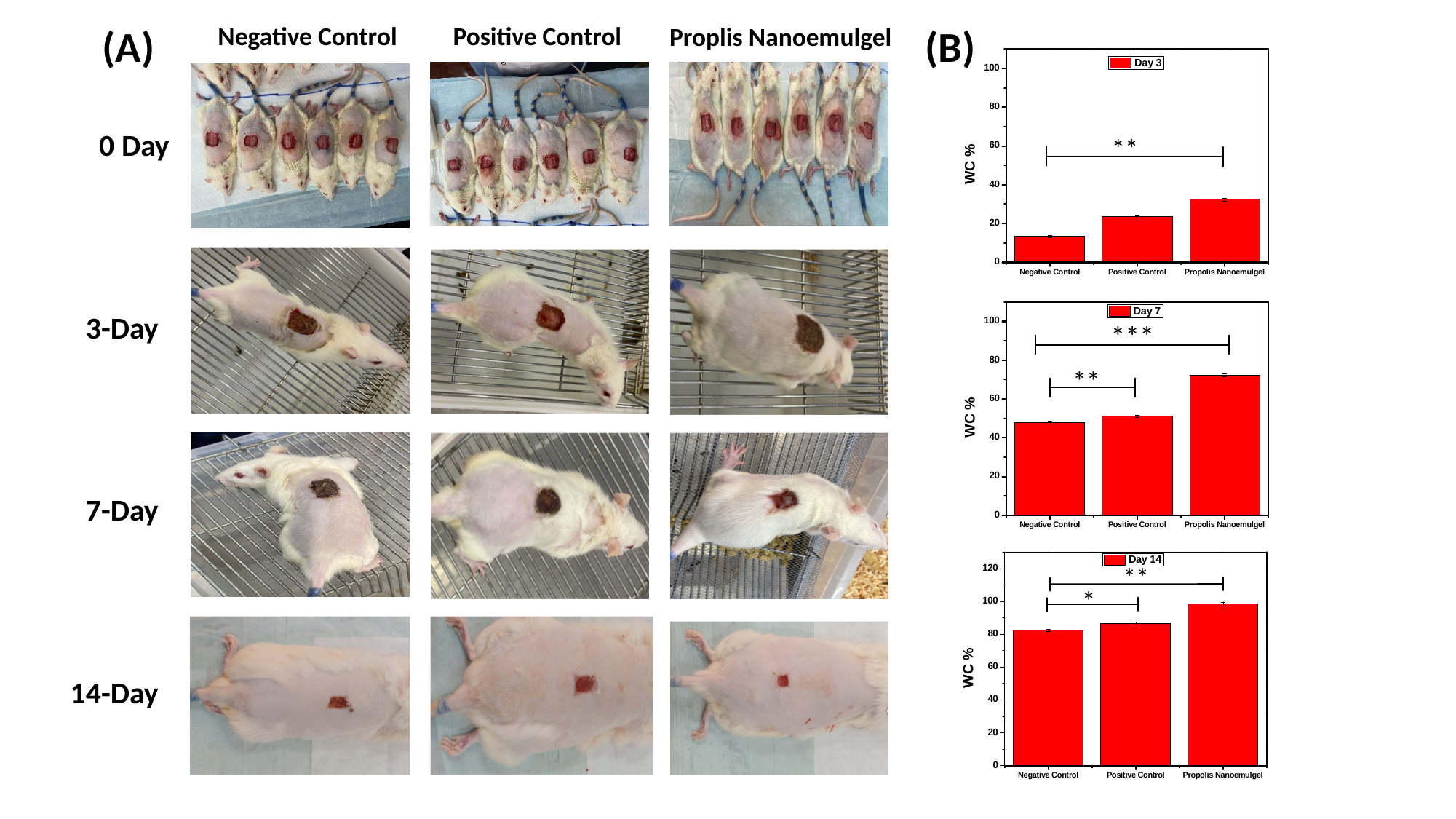

(A)
Negative Control
(B)
Positive Control
Proplis Nanoemulgel
0 Day
3-Day
7-Day
14-Day

Supplement: Supplementary file 4 — Supplementary Material 4 [file 41598_2026_50846_MOESM4_ESM.pptx]
